# Intraoperative and Anesthesia-Related Cardiac Arrest and Its Mortality in Older Patients: A 15-Year Survey in a Tertiary Teaching Hospital

**DOI:** 10.1371/journal.pone.0104041

**Published:** 2014-08-12

**Authors:** Juscimar C. Nunes, Jose R. C. Braz, Thais S. Oliveira, Lidia R. de Carvalho, Yara M. M. Castiglia, Leandro G. Braz

**Affiliations:** 1 Department of Anesthesiology, Botucatu Medical School, UNESP – Univ Estadual Paulista, Botucatu, São Paulo, Brazil; 2 Department of Biostatistics, Institute of Biosciences, UNESP – Univ Estadual Paulista, Botucatu, São Paulo, Brazil; San Raffaele Scientific Institute, Italy

## Abstract

**Background:**

Little information is known about factors that influence perioperative and anesthesia-related cardiac arrest (CA) in older patients. This study evaluated the incidence, causes and outcome of intraoperative and anesthesia-related CA in older patients in a Brazilian teaching hospital between 1996 and 2010.

**Methods:**

During the study, older patients received 18,367 anesthetics. Data collected included patient characteristics, surgical procedures, American Society of Anesthesiologists (ASA) physical status, anesthesia type, medical specialty team and outcome. All CAs were categorized by cause into one of four groups: patient's disease/condition-related, surgery-related, totally anesthesia-related or partially anesthesia-related.

**Results:**

All intraoperative CAs and deaths rates are shown per 10,000 anesthetics. There were 100 CAs (54.44; 95% confidence intervals [CI]: 44.68–64.20) and 68 deaths (37.02; 95% CI: 27.56–46.48). The majority of CAs were patient's disease-/condition-related (43.5; 95% CI: 13.44–73.68). There were six anesthesia-related CAs (3.26; 95% CI: 0.65–5.87) - 1 totally and 5 partially anesthesia-related, and three deaths, all partially anesthesia-related (1.63; 95% CI: 0.0–3.47). ASA I-II physical status patients presented no anesthesia-related CA. Anesthesia-related CA, absent in the last five years of the study, was due to medication-/airway-related causes. ASA physical status was the most important predictor of CA (odds ratio: 14.52; 95% CI: 4.48–47.08; *P*<0.001) followed by emergency surgery (odds ratio: 8.07; 95% CI: 5.14–12.68; *P*<0.001).

**Conclusions:**

The study identified high incidence of intraoperative CAs with high mortality in older patients. The large majority of CAs were caused by factors not anesthesia-related. Anesthesia-related CA and mortality rates were 3.26 and 1.63 per 10,000 anesthetics, with no anesthesia-related CA in the last five years of the study. Major predictors of intraoperative CAs were poorer ASA physical status and emergency surgery. All anesthesia-related CAs were medication-related or airway-related, which is important for prevention strategies.

## Introduction

The global population is ageing. According to the World Health Organization, people ≥60 years old in developing countries, such as Brazil, are considered elderly [1]. A significant proportion of older people are living with chronic diseases, such as cardiovascular disease, cognitive impairments and a reduced physiological reserve, which remain a challenge for all areas of medicine [2].

Few studies on perioperative cardiac arrest (CA) and mortality carried out exclusively in geriatric patients undergoing all types of surgery have been published [3]–[6]. However, there are no studies on anesthesia-related CA and death exclusively in elderly patients. Therefore, little information is known about factors that influence intraoperative and anesthesia-related CA and death in older surgical patients.

A survey performed from 1996 to 2005 in a Brazilian general tertiary teaching hospital [7] and other studies that included patients of all age groups [8], [9], found that the perioperative CA and mortality rates were higher in older adults than in young adults. Recognizing the importance of identification of high-risk patients to guide the planning, resourcing, and expert staffing required to enhance the safety and improve the anesthesia outcomes for older patients, our institution developed initiatives for the purpose of improving the care of surgical patients. This involved the organization of services for the care of older patients, including the operating room (OR) materials and equipments such as anesthesia workstations with ventilators to provide adequate ventilation and monitoring, and devices for temperature control, new drugs for anesthesia induction and increasing the number of adult intensive care beds.

The aim of this study was to evaluate the incidence, triggering factors, causes and outcome of intraoperative and anesthesia-related CA in older surgical patients over a 15-yr period in a Brazilian general tertiary teaching hospital.

## Methods

The project was approved by the Human Research Ethics Committee of the Botucatu Medical School (Ref: 3694-2010), who waived the requirement for written informed consent. This survey analyzed all reported CA in 18,367 consecutive anesthetics given to all older patients at least 60 years of age requiring anesthesia services at the Hospital of the Botucatu Medical School, *Universidade Estadual Paulista* (UNESP), São Paulo State, Brazil, a public tertiary teaching hospital, from January 1, 1996 to December 31, 2010. The UNESP hospital is a 450-bed tertiary-care referral facility with a catchment of 2 million people that performs approximately 7,000 surgeries per year on people of all age groups. The hospital has a 33-bed adult intensive care unit.

The basic safety monitoring protocol in the OR during regional and neuroaxial anesthesia and sedation included electrocardiogram, automatic non-invasive blood pressure and pulse oximetry. For general anesthesia were measured the core temperature, ventilation parameters, capnography and oxygen concentration. After 2005 the delivered nitrous oxide and halogenated anesthetic vapour concentrations were also measured. Critically ill patients received invasive blood pressure and central venous pressure monitoring. The same basic monitoring described for the OR was also used in the postanesthesia care unit (PACU).

Cardiac arrests and deaths in older patients in the OR and PACU were identified through an anesthesia database developed from a quality assessment form that is part of the mandatory documentation for each anesthetic procedure. The forms were completed by the anesthesia staff responsible for each administration of anesthetic. The recorded data included the date and location; patient characteristics; American Society of Anesthesiologists (ASA) physical status classification; surgical procedures (elective, urgent or emergency surgery); medical specialty team; anesthesia type (general, regional or monitored and supportive anesthesia care associated or not with local anesthesia in critically ill patients); outcome; and a 95-item checklist of airway, respiratory, cardiocirculatory, neurological, renal and miscellaneous events related to the OR or PACU stay.

Cardiac arrest was defined as the cessation of cardiac mechanical activity with loss of effective circulation determined by the absence of a palpable central pulse. The resuscitation was performed according to the interventions and protocols for Advanced Cardiovascular Life Support (ACLS) guidelines.

The anesthetist responsible for each case of CA was asked to review the case and provide a written summary for peer review. The medical and anesthesia records, the written summary, and, when applicable, the necropsy report were analyzed by the Anesthesia Cardiac Arrest Study Commission, which was composed of three authors who are faculty members in the Department of Anesthesiology (YMMC, JRCB and LGB). The triggering factors of CA and death were retrospectively assigned to one of four groups: (1) totally related to the surgery when a technical surgical problem was the only or the major contributory factor, (2) totally related to an older patient's disease or condition, (3) totally related to anesthesia when anesthesia was the only or the major contributory factor or (4) partially related to anesthesia when an older patient's condition/disease or surgical procedure was a factor additional to anesthesia in the CA or death. Unanimity was reached on the triggering factors in the majority of the CAs and deaths. Disagreements among the three members were resolved by discussion, and agreement was reached in all cases when at least two of the three members agreed on the triggering factors.

Some of the CAs in the current study were likely included in our previous study that examined perioperative CAs in all age groups from April 1996 to March 2005, but did not evaluate CAs in older patients exclusively [7]. Thus, only some of these older patients who experienced intraoperative CA (48) are included in the numerator in the present study whereas all of the older patients (6,796 who received anesthesia) are included in the denominator.

### Statistical analysis

The incidence (expressed per 10,000 anesthetics) and 95% confidence interval (CI) of intraoperative CA and mortality were calculated according to age, sex, ASA physical status, surgical procedures, anesthetic technique, surgical area and triggering factors. In these cases, the difference between rates were considered statistically significant if their respective 95% CIs did not overlap. Factors predicting intraoperative CA (age, sex, ASA physical status, surgical procedures and anesthesia technique) were analyzed with multivariate forward stepwise logistical regression with odds ratio and 95% CI reported. The conditional independence of all variables and stratifications was verified by the Cochran-Mantel-Haenszel test while the homogeneity of the odds ratio was validated by the Breslow-Day test. The statistical analysis was performed using the software Statistical Package for Social Sciences for Windows (version 17.1; SPSS Inc., Chicago, IL, USA). A *P* value<0.05 was considered statistically significant.

## Results

Over the 15 years of the study, 18,367 older patients were anesthetized. One hundred CAs (54.44 per 10,000 anesthetics; 95% CI: 44.68–64.20) and 68 deaths (37.02 per 10,000 anesthetics; 95% CI: 27.56–46.48) were identified within the intraoperative period (OR and PACU). Thus, only 32% of the older patients who experienced intraoperative CA survived. The majority of CAs and deaths occurred in the OR (93% and 94.12%, respectively) compared with the PACU (7% and 5.88%, respectively).

The intraoperative CA and death incidence in older surgical patients increased with the ASA physical status classification, in emergency surgery (12∶1 compared with elective surgery) (Table 1). General anesthesia (6.6∶1 compared with neuroaxial anesthesia) and an older patient's disease/condition were important factors of intraoperative CA (13.4∶1 compared with anesthesia and 5.7∶1 compared with surgery) and death (Tables 2 and 3, respectively). Multiclinical (two or more surgical areas) per 10,000 anesthetics (1034; 95% CI: 706–1,362), cardiac (412; 95% CI: 158–665), vascular (124; 95% CI: 61–185), thoracic (58; 95% CI: 24–91) or gastroenterological surgery (56; 95% CI: 10–102) were also important CA factors.

**Table 1:** Intraoperative cardiac arrest and death incidences among 18,367 anesthetics according to the characteristics of the older patients.

	Anesthetics	Cardiac Arrests			Deaths		
	*n*	*n*	Incidence per 10,000	95% CI	*n*	Incidence per 10,000	95% CI
**Age, yr**							
60–74	13,546	71	52.41	40.25–64.57	48	35.43	25.42–45.44
75–90	4,586	28	61.05	38.50–83.60	19	41.43	22.84–60.02
≥91	235	1	42.55	0.00–125.77	1	42.55	0.00–125.77
**Sex**							
Male	9,716	56	57.64	36.07–79.21	35	36.02	24.11–47.93
Female	8,651	44	50.86	28.42–73.30	33	38.15	25.16–51.14
**ASA physical status**							
I	1,836	1	5.45	0.00–20.66	1	5.45	0.00–16.13
II	8,975	2	2.22	0.00–6.87	1	1.11	0.00–3.29
III	5,754	23	39.97	17.10–62.84	16	27.80	14.20–41.40
IV	1,660	54	325.30	194.81–455.79	36	216.86	146.80–286.94
V	142	20	1408.45	650.88–2166.02	14	985.91	495.58–1476.24
**Surgical procedures**							
Elective	11,531	27	23.41	10.78–36.04	21	18.21	10.43–25.99
Urgent	4,610	10	21.69	1.72–41.66	5	10.84	1.34–20.34
Emergency	2,226	63	283.01	173.32–392.70	42	188.67	132.15–245.19

CI = confidence interval.

**Table 2:** Intraoperative cardiac arrest and death incidences among 18,367 anesthetics in older patients according to anesthesia technique.

	Anesthetics	Cardiac Arrests			Deaths		
Anesthesia technique	*n*	*n*	Incidence per 10,000	95% CI	*n*	Incidence per 10,000	95% CI
General anesthesia	10,541	86	81.58	56.70–106.48	57	54.07	40.07–68.07
Regional anesthesia							
- Epidural/spinal	6,520	8	12.27	0.04–24.50	5	7.66	0.95–14.39
- Plexus block	597	0	0.00	0.00–0.00	0	0.00	0.00–0.00
Sedation	488	0	0.00	0.00–0.00	0	0.00	0.00–0.00
Other#	221	6	271.49	10.54–532.44	6	271.49	57.22–485.76

CI = confidence interval;

# Monitored anesthesia and supportive care in ASA IV-V physical status patients.

**Table 3:** Intraoperative cardiac arrest and death incidences among 18,367 anesthetics in older patients according to triggering factor.

	Cardiac Arrests			Deaths		
Triggering factor	n	Incidence per 10,000	95% CI	n	Incidence per 10,000	95% CI
Patient's disease/condition	80	43.55	13.44–73.68	55	29.94	22.05–37.85
Surgery	14	7.62	3.63–11.61	10	5.44	2.07–8.81
Anesthesia	6	3.26	0.65–5.87	3	1.63	0.00–3.4
- Totally-related	1	0.54	-	0	0.00	-
- Partially-related	5	2.72	-	3	1.63	-

CI = confidence interval.

Poorer ASA physical status (III–V), emergency surgery and general anesthesia were significant predictive factors of intraoperative CA and death in older patient (Tables 4 and 5, respectively). However, age and sex were not predictors of intraoperative CA or mortality (Tables 4 and 5, respectively).

**Table 4:** Factors associated with intraoperative cardiac arrest in older patients.

Factors	Odds Ratio	95% CI	Estimated Coefficient	*P* value
**Age, yr**				
60–74	1.0 (Reference)			
75–90	0.89	0.57–1.42	−0.10	0.65
≥91	0.70	0.09–5.27	−0.35	0.73
**Sex**				
Male	1.0 (Reference)			
Female	1.01	0.67–1.52	0.10	0.96
**ASA physical status**				
I–II	1.0 (Reference)			
III–V	14.52	4.48–47.08	2.67	<0.001
**Surgical procedures**				
Nonemergency	1.0 (Reference)			
Emergency	8.07	5.14–12.68	2.09	<0.001
**Anesthesia technique**				
No general anesthesia	1.0 (Reference)			
General anesthesia	4.31	2.21–8.41	1.46	<0.001

ASA = American Society of Anesthesiologists; OR: odds ratio; CI = confidence interval.

**Table 5:** Factors associated with intraoperative mortality in older patients.

Factors	Odds Ratio	95% CI	Estimated Coefficient	*P* value
**Age, yr**				
60–74	1.0 (Reference)			
75–90	0.91	0.56–1.59	−0.09	0.75
≥91	1.07	0.14–8.16	0.07	0.97
**Sex**				
Male	1.0 (Reference)			
Female	0.858	0.52–1.40	−0.15	0.54
**ASA physical status**				
I–II	1.0 (Reference)			
III–V	13.34	3.15–56.47	2.59	<0.001
**Surgical procedures**				
Nonemergency	1.0 (Reference)			
Emergency	7.14	4.16–12.26	1.97	<0.001
**Anesthesia technique**				
No general anesthesia	1.0 (Reference)			
General anesthesia	4.06	1.86–8.85	1.40	<0.001

ASA = American Society of Anesthesiologists; OR: odds ratio; CI = confidence interval.

Anesthesia-related CA was considered a contributing factor in 5 cases and a primary factor in 1 case. Furthermore, anesthesia was considered a contributing factor in all 3 anesthesia-related deaths (Table 3). With regard to the triggering factors, 50% of the older patients who experienced anesthesia-related CAs survived, compared with 28.6% in the cases of surgery-related CAs and 31.25% in those of patient condition-/disease-related CAs. All anesthesia-related CAs and deaths in older surgical patients occurred from 1996 to 2005 at rates of 5.42 (95% CI: 1.2–9.6) and 2.7 (95% CI: 0.0–5.7) per 10,000 anesthetics, respectively, against CA and mortality rates of zero per 10,000 anesthetics in the 2006–2010 period.

Additional information on the 94 intraoperative CAs and 65 deaths attributable to an older patient's disease/condition or surgery factors is provided in Table 6. Sepsis and multiple organ failure were the most common causes of CA, while a ruptured aneurysm was the most common cause of mortality.

**Table 6:** Causes of intraoperative cardiac arrests and deaths attributed to an older patient's disease/condition or surgical factors.

	Cardiac arrests		Deaths		Mortality
Cause	*n*	%	*n*	%	%
Sepsis and multiple organ failure	29	30.85	14	21.53	48.27
Ruptured aneurysm: abdominal, thoracic or cerebral	21	22.34	19	29.23	90.48
Complications associated with cardiac surgery including inability to wean from cardiopulmonary bypass	19	20.22	15	23.08	78.95
Intraoperative myocardial infarction	6	6.38	4	6.15	66.66
Trauma: motor vehicle accident, gunshot wound or stab wound with exsanguinating hemorrhage	5	5.32	4	6.15	80.00
Exsanguinating hemorrhage during surgery associated with primary disease	5	5.32	3	4.62	60.00
Pulmonary embolus	3	3.19	3	4.62	100.0
Complications associated with radical cancer surgery	3	3.19	2	3.08	66.66
Technical surgical complications	3	3.19	1	1.54	33.34

Medication-related adverse events accounted for four (66.66%) anesthesia-related CAs, three of which were caused by cardiovascular collapse after neuroaxial anesthesia. Of these three patients, two ASA physical status IV older patients passed away in the OR during vascular surgery (lower limb amputation) while undergoing continuous epidural anesthesia. In the third patient, a 63-year-old male with an ASA physical status of III who suffered a femur fracture in a motor vehicle accident, and had multiple rib fractures, the CA occurred after the administration of a spinal anesthetic with hyperbaric bupivacaine (20 mg). Immediately after anesthesia induction, he developed hypotension and bradycardia, followed by cardiovascular collapse and CA. In this case, CA was attributed entirely to the anesthesia. Fortunately, the patient was successfully resuscitated and recovered completely. There was 1 case of fluid overload followed by acute pulmonary edema in the PACU after general anesthesia in a female patient with an ASA physical status of III and a medical history of chronic renal failure. This patient passed away.

Respiratory events accounted for two (33.33%) anesthesia-related CAs. In both cases, there was loss of airway and difficulty to intubate while the patients had significant underlying disease with an ASA physical status of III or IV. One patient with chronic obstructive pulmonary disease and *diabetes mellitus* had a cervical abscess and mediastinitis; intubation and ventilation were difficult after general anesthesia induction followed by hypoxemia, cardiovascular collapse and CA. The patient was successfully resuscitated and recovered. In another case, a patient, with atrial fibrillation after thoracoscopy to drain a pleural effusion, undergoing general anesthesia presented hypoventilation after tracheal extubation in the PACU followed by a difficult intubation, hypoxemia and CA. The patient was successfully resuscitated and recovered with myocardial infarction.

## Discussion

The main findings in this study were: (i) older patients continued to present high incidence of intraoperative CA with high mortality; (ii) anesthesia-related CA and mortality rates were 3.26 and 1.63 per 10,000 anesthetics, respectively; (iii) there was no anesthesia-related CA in ASA I–II physical status patients; (iv) there were no anesthesia-related CA and deaths rates in the last five years of the study; (v) the large majority of CAs (94%) and deaths (95.5%) that occurred during anesthesia were caused by factors not anesthesia-related, and (vi) ASA physical status and emergency surgery, but not age and sex, were significant predictors of intraoperative CA and death in older patients.

A study conducted in Thailand in older surgical patients reported a 24-hour perioperative death rate of 39.33 per 10,000 anesthetics [5]. Another Thai study reported 24-hour perioperative CA and death rates of 40.42 and 31.44 per 10,000 anesthetics, respectively, in older patients who underwent non-cardiac surgery [3]. A previous survey of the perioperative CA rate in all age groups conducted at our institution revealed a higher incidence of perioperative CA in older patients of 70.62 per 10,000 anesthetics [7]. A few other studies conducted in developed countries in the last decade reported lower perioperative CA rates ranging from 10.73 to 16.08 per 10,000 anesthetics in older surgical patients [9], [10]. A systematic review [11] and a meta-analysis [12] of the literature verified that perioperative mortality rates reported in surgical patients of all age groups were higher in developing than in developed countries.

It is difficult to compare the intraoperative CA and mortality rates in our study with the results of previous studies (Table 7) because of considerable differences in the methods and patient populations among studies [3], [5]–[10], [13]–[16]. There is no consensus in the literature regarding the definition of perioperative and anesthesia-related mortality [17].

**Table 7:** Previous studies of perioperative and anesthesia-related cardiac arrest and mortality in older patients.

Investigators and publication	Time period and data source	Number of patients Time of CA/death	Age (yr)	CA incidence per 10,000 anesthetics		Mortality incidence per 10,000 anesthetics	
				Perioperative	Anesthesia-related	Perioperative	Anesthesia-related
**Marx et al. ** [14]	1965–1969	4,176	**>60**	**NR**	**NR**	**514.84**	**35.91**
**1973**	Teaching hospital	Death within 7 days	61–70			442.02	20.40
	USA		71–80			679.86	34.23
			>81			821.91	684.93
**Olsson & Hallen ** [13] **]**	1967–1984	NR	**>60**	**NR**	**7.1**	**NR**	**NR**
**1988**	Teaching hospital	CA and death in OR					
	Sweden	Excluded: cardiac surgery					
**Aubas et al. ** [15]	1983–1987	34,633	**≥55**	**NR**	**4.33**	**NR**	**1.44**
**1991**	Teaching hospital	CA and deaths in OR and PACU	55–74		2.67		1.14
	France		75–84		8.86		2.95
			≥85		12.04		0
**Kubota et al. ** [16]	1962–1992	15,351	**≥65**	**NR**	**0.65**	**NR**	**0**
**1994**	Teaching hospital	CA and death in OR	65–70		0		0
	Japan	Excluded: cardiac surgery	70–80		1.66		0
		transplant cases					
			80–90		0		0
			90–95		0		0
**Biboulet et al. ** [8]	1989–1995	28,987	**≥55**	**NR**	**2.75**	**NR**	**1.72**
**2001**	Teaching hospital	CA and death within 12 h	55–74		1.86		0.46
	France	Excluded: ASA V patients	75–84		1.83		1.83
			>84		14.36		14.36
**Kawashima et al. ** [10] **]**	1999	208,568	**≥66**	**10.73**	**NR**	**NR**	**0.33**
**2002**	Group of hospitals	CA and death within 7 days	66–85	11.02		6.56	0.25
	Japan		≥86	6.66		5.18	1.48
**Braz et al. ** [7]	1996–2005	6,796	**≥65**	**70.62**	**5.88**	**NR**	**2.94**
**2006**	Teaching hospital	CA and death in OR and PACU	65–79	68.78	5.29		1.76
	Brazil		≥80	79.33	8.88		8.88
							
* **Rodanant et al. ** [5]	2003–2004	23,899	**≥65**	**NR**	**NR**	**39.33**	**NR**
**2007**	Group of hospitals	Deaths within 24 h					
	Thailand						
* **Tamdee et al. ** [3]	2003–2007	8,905	**≥65**	**40.42**	**NR**	**31.44**	**NR**
**2009**	Teaching hospital	CA and death within 24 h	65–75	22.87			
	Thailand	Excluded: cardiac surgery	76–85	67.22			
			>86	148.51			
* **Story et al. ** [6]	2004, 2007–2008	4,158	**≥70**	**NR**	**NR**	**519.48**	**NR**
**2010**	Group of hospitals	Deaths within 30 days	70–79			355.4	
	Australia, New Zealand	Excluded: cardiac surgery	80–89			695.6	
			≥90			1219.5	
**Goswani et al. ** [9]	2005 – 2007	223,961	**≥70**	**16.08**	**NR**	**NR**	**NR**
**2012**	Group of hospitals	CA in OR					
	USA	Excluded: cardiac surgery					
		peripheral nerve block					
		trauma cases					
		transplant cases					
**Current study**	1996–2010	18,367	**≥60**	**54.44**	**3.26**	**37.02**	**1.63**
	Teaching hospital	CA and death in OR and PACU	60–74	52.41	2.95	35.43	1.47
	Brazil		75–90	61.05	4.36	41.43	2.18
			≥91	42.55	0	42.55	0

*Studies that included only older patients; CA: cardiac arrest; NR: not reported; OR: operating room; ASA = American Society of Anesthesiologists; PACU: postanesthesia care unit.

Some perioperative CA studies excluded ASA V older patients [8], cardiac surgery [3], [6], [9], [13], [16] or trauma [9] and transplant surgeries [9], [16]. Therefore, the inclusion of both all surgery types and poorer ASA physical status patients, resulting from certain pre-existing morbidities, certainly influenced our CA and death rates. Ninety-seven percent of the intraoperative CAs occurred in poorer ASA physical status (III–V) patients with sepsis and multiple organ failure being the most important causes (30.8%). Sepsis occurs at a high incidence in Brazil and other developing countries with high mortality [18].

The elderly are more predisposed to vascular disease than young persons. Thus, a ruptured aneurysm, or complications associated with cardiac surgery or intraoperative myocardial infarction were also important causes of intraoperative CA and death attributed to an older patient's disease/condition or surgical factors in the current study. Our study demonstrated that patients presenting a poorer ASA physical status (III–V) were more likely (14-fold) to suffer intraoperative CA than ASA I–II patients. Previous studies have shown a similar association of perioperative CA and mortality with poorer ASA physical status in the older individuals [3], [5], [6]. In our study, it was verified that many older patients arrived at the OR in poor health. Intraoperative CAs and deaths that occurred in some of these patients might have been prevented by an adequate preoperative assessment. A study showed that perioperative death might have been prevented by anesthesia care in approximately 1 per 13,900 anesthetics [19]. These findings demonstrate a persistent need to improve the quantity and quality of resource utilization and access to healthcare, which are inadequate in Brazil and in other developing countries. In addition, it is necessary to adopt perioperative medical practices with demonstrable effectiveness, organize multidisciplinary discussion of adverse effects and implement of evidence-based safety protocols to improve perioperative patient care [20], [21].

As demonstrated in our study and in others [3], [5], [6], the urgency of surgery is a well-established preoperative predictive factor for intraoperative CA and mortality in older patients. The risk of CA in emergency surgery is likely due to many factors, such as the impossibility of adequate evaluation and optimization of the patients before the surgery.

Despite the differences in methodology, studies [6]–[10],[15] have suggested that anesthesia-related CA and mortality rates in older people are lower today than they were 40 years ago (35.91 per 10,000 anesthetics) [14]. Most studies performed in the last two decades in developed countries have reported in older patients anesthesia-related death rates ranging from 0.00 to 1.72 per 10,000 anesthetics [8], [15], [19], which represents an improvement of almost twenty-fold. Our study confirmed this trend (i.e., 1.63 anesthesia-related deaths per 10,000 anesthetics in its entire timeframe and zero per 10,000 anesthetics in its last five years in older patients). These data may be associated with the improvements achieved in the geriatric anesthesia safety in our institution in the recent years.

In contrast to our current study, many prior works have identified advanced age (>75 yr) as a risk factor for perioperative CA [3], [6], [8], [15], and also did not find age itself to be an independent risk factor [10].

Sex was not a risk factor for intraoperative CA in our study. Sex is known to be a significant risk factor for perioperative CA and mortality in young men suffering from trauma [7]. In our study, trauma was the fifth highest cause of intraoperative CA and death in older surgical patients. The elderly are less predisposed to trauma and violence than young persons.

Our study showed that general anesthesia was a predictor of intraoperative CA. However, this may be attributable to the fact that many high-risk surgeries (cardiac, thoracic, vascular, gastroenterological) in older patients were performed under general anesthesia. Another possible confounding factor in this case is that the anesthesiologist often prefers to give general anesthesia to the most fragile patients, independently of the type of surgery.

In our study, respiratory causes of anesthesia-related CA were less frequent (33%) than medication causes (67%). In a recent study in all age groups [22], the authors verified that 64% of anesthesia-related CAs were caused by airway complications. There were three cases of CA after the administration of neuroaxial anesthesia. After neuroaxial anesthesia, a condition in which the loss of sympathetically mediated vasoconstriction may decrease vascular resistance, and the loss of sympathetically mediated cardiac stimulation may diminish both the heart rate and stroke volume. The vasodilatation may permit the peripheral pooling of blood and lower the end-diastolic volume, stroke volume and cardiac output in older patients [23]. In the last five years of our study, we preferentially used isobaric bupivacaine for spinal anesthesia in older patients. This local anesthetic determines lower levels of sympathetic block with lower cardiovascular effects than hyperbaric bupivacaine in older patients [24].

There may have been some methodological weaknesses in our study. First, the data are derived from adverse events reported by faculty and residents. Underreporting is likely in this situation, even though filling out the form for each case was mandatory. To minimize the risk of underreporting CAs and deaths, the information was cross-checked with the operating theater records and hospital administration. Second, this study is representative of the experience in one tertiary referral center. Practices specific to our institution may have influenced our results and may not represent the entire spectrum of elderly patient care practices. A multi-institutional anesthesia database would not only provide useful insights into injury mechanisms, but would also facilitate the development of universally applicable strategies [25].

## Conclusions

Our study has identified high incidence of intraoperative CA with high mortality in older patients. The majority of CAs and deaths were caused by factors not related to anesthesia. Anesthesia-related CA and death rates were 3.26 and 1.63 per 10,000 anesthetics, respectively. Improvements achieved in anesthesia safety in the older patients in our institution allowed no anesthesia-related CA and mortality in the last five years of the study and no anesthesia-related CA in ASA I-II physical status older patients. Major predictor of CA and death was a preoperative indicator of health, the ASA physical status, followed by emergency surgery. The fact that all anesthesia-related CAs were associated with medication administration or airway management is important for the development of prevention strategies. The high fatality rate in older surgical patients due to poor physical status suggests that primary prevention might be the key to reducing mortality from intraoperative CA. These findings indicate the need to improve medical perioperative practices for older people with highly complex medical problems in a demonstrably effective manner in under-resourced settings.
